# Dutch–Flemish translation of nine pediatric item banks from the Patient-Reported Outcomes Measurement Information System (PROMIS)^®^

**DOI:** 10.1007/s11136-015-0966-y

**Published:** 2015-03-28

**Authors:** Lotte Haverman, Martha A. Grootenhuis, Hein Raat, Marion A. J. van Rossum, Eline van Dulmen-den Broeder, Karel Hoppenbrouwers, Helena Correia, David Cella, Leo D. Roorda, Caroline B. Terwee

**Affiliations:** 1 Psychosocial Department, Emma Children’s Hospital, Academic Medical Center, Meibergdreef 9, 1105 AZ Amsterdam, The Netherlands; 2 Department of Public Health, Erasmus MC, University Medical Center, Wytemaweg 80, 3015 CN Rotterdam, The Netherlands; 3 Department of Pediatric Hematology, Immunology and Infectious Diseases, Emma Children’s Hospital, Academic Medical Center, Meibergdreef 9, 1105 AZ Amsterdam, The Netherlands; 4 Department of Pediatric Rheumatology, Jan van Breemen Research Institute | Reade, Dr. Jan van Breemenstraat 2, 1056 AB Amsterdam, The Netherlands; 5 Department of Pediatrics, VU University Medical Center, De Boelelaan 1117, 1081 HV Amsterdam, The Netherlands; 6 Centre for Environment and Health, Youth Health Care, KU Leuven, Kapucijnenvoer 35, 3000 Louvain, Belgium; 7 Department of Medical Social Sciences, Northwestern University Feinberg School of Medicine, 633 N. Saint Clair St., Chicago, IL 60611 USA; 8 Center on Outcomes Research and Education, Evanston Northwestern Healthcare, 1001 University Pl, Suite 100, Evanston, IL 60201 USA; 9 Amsterdam Rehabilitation Research Center | Reade, Overtoom 283, 1054 HW Amsterdam, The Netherlands; 10 Department of Epidemiology and Biostatistics and the EMGO Institute for Health and Care Research, VU University Medical Center, De Boelelaan 1117, 1081 HV Amsterdam, The Netherlands

**Keywords:** Quality improvement, PROMIS, Pediatric item banks, HRQOL, Measurements, PROs

## Abstract

**Purpose:**

The Patient-Reported Outcomes Measurement Information System (PROMIS^®^) is a new, state-of-the-art assessment system for measuring patient-reported health and well-being of adults and children. It has the potential to be more valid, reliable, and responsive than existing PROMs. The items banks are designed to be self-reported and completed by children aged 8–18 years. The PROMIS items can be administered in short forms or through computerized adaptive testing. This paper describes the translation and cultural adaption of nine PROMIS item banks (151 items) for children in Dutch–Flemish.

**Methods:**

The translation was performed by FACITtrans using standardized PROMIS methodology and approved by the PROMIS Statistical Center. The translation included four forward translations, two back-translations, three independent reviews (at least two Dutch, one Flemish), and pretesting in 24 children from the Netherlands and Flanders.

**Results:**

For some items, it was necessary to have separate translations for Dutch and Flemish: physical function—mobility (three items), anger (one item), pain interference (two items), and asthma impact (one item). Challenges faced in the translation process included scarcity or overabundance of possible translations, unclear item descriptions, constructs broader/smaller in the target language, difficulties in rank ordering items, differences in unit of measurement, irrelevant items, or differences in performance of activities. By addressing these challenges, acceptable translations were obtained for all items.

**Conclusion:**

The Dutch–Flemish PROMIS items are linguistically equivalent to the original USA version. Short forms are now available for use, and entire item banks are ready for cross-cultural validation in the Netherlands and Flanders.

## Background

The use of pediatric Patient-Reported Outcome Measures (PROMs) in pediatric research, clinical trials, and clinical practice has increased in the past years to obtain insight into the consequences of a chronic illness on a child’s life, to monitor patient’s health status and to assist with communication [1].

As summarized by Terwee et al. [2], PROMs are not free of problems and challenges. For example, for many constructs, several instruments of varying quality have been developed. Some PROMs are burdensome for patients because they are too long, or contain irrelevant, incomprehensible or poorly formulated questions. Therefore, the Patient-Reported Outcomes Measurement Information System (PROMIS) project was initiated to advance the science and application of PROMs [3, 4]. The PROMIS project transformed existing PROMs into a more optimal assessment system for measuring health-related quality of life (HRQOL). This new system has shown to have two important advantages. Firstly, the system has a higher validity, reliability, and better responsiveness than the existing PROMs [5–7]. Secondly, patients need to answer fewer items compared to traditional questionnaires, because the PROMIS consists of a set of item banks. An item bank is a set of questions that have all been statistically calibrated to the same underlying construct. The items from an item bank can be administered in brief, fixed questionnaires, or more efficiently through computerized adaptive testing (CAT) [8]. A CAT is a computer-administered test in that, after the first item, the presentation of items is determined by a person’s response to previous ones. After about 5–7 items, the computer stops presenting questions [9].

The pediatric PROMIS project focused on the development of self-report item banks across several health domains for youth aged 8–18 years. The primary focus was on the measurement of generic health domains that are important for children across a variety of illnesses [10–15].

It is expected that PROMIS will be implemented worldwide, and that PROMIS instruments will rapidly replace existing PROMs [16–18]. In 2011, the Dutch–Flemish pediatric PROMIS Group was established with the aim to implement pediatric PROMIS instruments in the Netherlands and Flanders (northern Belgium). We strived to obtain one uniform Dutch–Flemish translation for all items. The goal of a universal approach to translations was to result in one version for multiple countries instead of country-specific versions of the same language. This approach was chosen for practical reasons and to avoid unnecessary language bias introduced by multiple translations. There is also policy support for one official language [19]. The first step concerned the translation and cultural adaptation of the PROMIS items into Dutch–Flemish. This paper describes the translation of nine item banks for children.

## Methods

The translation was obtained using a universal approach based on the established Functional Assessment of Chronic Illness Therapy (FACIT) Multilingual Translation Methodology [20, 21]. The goal of this methodology was to attain five dimensions of cross-cultural equivalence:

1. Semantic/linguistic: The meaning of the item is the same in the source and translated language;
2. Content: The item is relevant to both cultures;
3. Conceptual: The translated document measures the same theoretical constructs as the source;
4. Criterion: When compared to a known or standardized measurement, the translation exhibits similar measurement properties of the source;
5. Technical: The method of assessment results in comparable measurements in both cultures [22].

We addressed the first three dimensions. The last two will need to be checked by additional psychometric validation. We strived to obtain one uniform Dutch–Flemish translation for all items, but separate translations for Dutch and Flemish were produced when necessary.

### Translation

The translation team implemented specific steps in order to develop precise and culturally appropriate translations based on the English source. The steps involved are itemized below:

1. Four simultaneous forward translations: Source items in English were translated into Dutch–Flemish by four native Dutch- or Flemish-speaking, independent professional translators (two from the Netherlands, two from Flanders) experienced in the field of PROM survey research;
2. Reconciled single Dutch–Flemish translation: A fifth independent translator, a native Dutch-speaking professional from the Netherlands, reconciled the four forward translations by choosing the better of the forward translations and resolved discrepancies between them;
3. Back-translation: This reconciled version was then back-translated by two English-speaking translators, one fluent in Dutch and one in Flemish, experienced in the field of PROM survey research. The back-translators were blind to the English source version;
4. Back-translation review: FACITtrans staff compared source and back-translated English versions to identify discrepancies in the back-translations and provided clarification to the reviewers on the intent behind the items;
5. Expert reviews: Three to four bilingual experts from the Dutch–Flemish pediatric PROMIS Group (at least three Dutch and one Flemish) examined all of the preceding steps and selected the most appropriate translation for each item or provided alternate translations if the previous translations were not acceptable;
6. Pre-finalization review: FACITtrans staff evaluated the merit of the reviewer’s comments, identified potential problems in their recommended translations, and formulated questions and comments to guide the Dutch–Flemish language coordinator;
7. Finalization: The Dutch–Flemish language coordinator, who worked on the translation development, determined the final translation by reviewing all the information and addressing FACITtrans staff’s comments. Along with the final translation, the language coordinator also provided literal back-translation and polished back-translation;
8. Harmonization and quality assurance: FACITtrans staff assessed the equivalence of the final translation and verified that documentation of the decision-making process was complete. The Dutch language coordinator was consulted again for additional input when necessary;
9. Formatting, typesetting, and proofreading of the final questionnaire or item forms were performed by two proofreaders working independently.

### Testing of translations

The target language version resulting from the described translation process was pretested in a pilot study in a convenience sample of native Dutch- or Flemish-speaking children aged 8–17 years. Exclusion criteria were (1) unable to read and speak Dutch or Flemish and (2) unable to provide verbal informed consent. Each item was debriefed using a standardized cognitive debriefing interview to ensure that the meaning of the item remained equivalent after translation. After the interviews, FACITtrans staff analyzed subjects’ comments to empirically determine the linguistic validity and acceptability of the questionnaire.

## Results

### Translation

- *Differences between Dutch and Flemish* For four out of nine item banks (Table 1) and seven out of 151 items (5 %), it was necessary to have separate translations for Dutch and Flemish for some items (see asterisks Table 1). For example, the word “walking” was translated as “lopen” in Dutch, but had to be translated as “stappen” in Flemish because “lopen” means running in Flemish (“hardlopen” in Dutch) and “stappen” means going out in Dutch.

**Table 1:** Translated pediatric PROMIS item banks

English	Dutch–Flemish	Number of items
Asthma impact^a^	Astma impact	17
Anger^a^	Boosheid	5
Anxiety	Angst	13
Depressive symptoms	Depressieve klachten	13
Fatigue	Vermoeidheid	23
Physical function—mobility^c^	Lichamelijk functioneren—mobiliteit	23
Physical function—upper Extremity	Lichamelijk functioneren—bovenste extremiteit	29
Pain interference^b^	Belemmeringen door pijn	13
Peer relationships	Relaties met leeftijdsgenoten	15
Total	Total	151

The number of items with a Dutch and Flemish version; a = 1, b = 2 and c = 3
- *Different measurements* The measurement units used in the Netherlands and Flanders are different from those used in the USA. For example, in the Netherlands and Flanders, kilos and meters are used instead of pounds and miles.
- *Different concepts* The item “I worried when I went to bed at night” was translated as “Ik maakte me zorgen als ik ‘s avonds naar bed ging” (I worried when I went to bed in the evening). Dutch distinguishes between “avond” (evening—before midnight) and “nacht” (night—after midnight).
- *Absence of literal translations* The item “I could walk across the room,” was translated as “Ik kon naar de andere kant van de kamer lopen” (I could walk to the other side of the room). There is no literal translation for “across the room.”
- *Different ways of saying* The items “I could put on my socks by myself” and “I could put on my shoes by myself” have two different translations in Dutch, in Dutch you say to pull on socks and put on shoes.

### Pilot testing

The Dutch–Flemish PROMIS item banks were tested in the Netherlands and Flanders. In total, 24 children (12 from Belgium, 12 from the Netherlands) participated, of which 58 % were female (*n* = 14) and 42 % were male (*n* = 10), with an average age of 14 years. All participants were from the general population, with the exception of six children recruited for the Peer Relationships and Asthma Impact item bank, who were all diagnosed with asthma. Five slight changes in the wording of questions were made after testing. For example, the phrase “Ik voelde me aanvaard door andere kinderen” was changed to “Ik voelde me geaccepteerd door andere kinderen…” in which “aanvaard” as well as “geaccepteerd” both means accepted.

## Discussion

Nine pediatric PROMIS item banks for children were translated from English into Dutch–Flemish. Some difficulties were found in the translation of some items, but eventually an acceptable translation was obtained for all items.

The translation was produced using a strong standard PROMIS methodology. The PROMIS translation methodology was developed through substantial research in the HRQOL field to ensure that translations reflect conceptual equivalence with the English source and are rendered in language that is culturally acceptable and relevant to the target population. We strived to obtain one uniform Dutch–Flemish translation for all items. Nevertheless, for seven items, it was necessary to have separate translations for Dutch and Flemish.

The use of pediatric PROMIS item banks has clear advantages over traditional PROMs [23], and therefore, we highly recommend to use these PROMIS instruments. PROMIS instruments have been found to have less measurement error and better responsiveness than existing measures, which leads to smaller sample sizes required in clinical studies [24, 25]. PROMIS scores are easy by using item response theory (IRT) methods; scores on interval level are obtained. In addition, all PROMIS instruments are scored on a common metric: scores are expressed as T-scores with a mean score of 50 (representing the mean score of the reference population) and a standard deviation of 10.

PROMIS instruments can be administered in short forms or through CAT (or a combination of both). CAT has great advantages over traditional paper questionnaires. In children, only one study has been performed with mixed results, so more studies are needed [25].

A limitation of this study is that the questionnaires are translated to Dutch–Flemish, but the content validity of the items banks was not yet analyzed. Before PROMIS can be considered valid for use in the Netherlands and Flanders, several steps should ideally be taken, as described by Terwee et al. [2]. Cross-cultural validation studies are needed to evaluate the IRT model fit in Dutch–Flemish children and to test for possible differential item functioning (DIF) between language groups. For example, the use of different units of measurement may have affected the item difficulty and may therefore introduce DIF. All persons at a given trait level should answer an item in the same way regardless of the language version completed. If an item functions differently in the original and translated versions, the item exhibits DIF with regard to translation. If important language DIF is found, language-specific item calibrations may need to be developed. Further research on these translations will increase confidence in their use. To make the first step in validating the pediatric item banks in the Netherlands, we will start a psychometric study in children with Juvenile Idiopathic Arthritis.

In conclusion, the translated versions of the Dutch PROMIS items are linguistically acceptable and are ready for cross-cultural validation studies in the Netherlands and Flanders.

## References

[1] Snyder CF, Aaronson NK, Choucair AK, Elliott TE, Greenhalgh J, Halyard MY (2012). Implementing patient-reported outcomes assessment in clinical practice: A review of the options and considerations. Quality of Life Research.

[2] Terwee, C.B., Roorda, L.D., de Vet, H.C., Dekker, J., Westhovens, R., van Leeuwen, J., et al. (2014). Dutch–Flemish translation of 17 item banks from the Patient-Reported Outcomes Measurement Information System (PROMIS). *Quality of Life Research*, 23(6), 1733–1741. 10.1007/s11136-013-0611-6 24402179

[3] Cella D, Yount S, Rothrock N, Gershon R, Cook K, Reeve B (2007). The Patient-Reported Outcomes Measurement Information System (PROMIS): Progress of an NIH roadmap cooperative group during its first two years. Medical Care.

[4] Cella D, Riley W, Stone A, Rothrock N, Reeve B, Yount S (2010). The Patient-Reported Outcomes Measurement Information System (PROMIS) developed and tested its first wave of adult self-reported health outcome item banks: 2005–2008. Journal of Clinical Epidemiology.

[5] Fries J, Rose M, Krishnan E (2011). The PROMIS of better outcome assessment: Responsiveness, floor and ceiling effects, and internet administration. The Journal of Rheumatology.

[6] Magasi S, Ryan G, Revicki D, Lenderking W, Hays RD, Brod M (2012). Content validity of patient-reported outcome measures: Perspectives from a PROMIS meeting. Quality of Life Research.

[7] Fries JF, Krishnan E, Rose M, Lingala B, Bruce B (2011). Improved responsiveness and reduced sample size requirements of PROMIS physical function scales with item response theory. Arthritis Research Therapy.

[8] Reeve BB, Hays RD, Bjorner JB, Cook KF, Crane PK, Teresi JA (2007). Psychometric evaluation and calibration of health-related quality of life item banks: Plans for the Patient-Reported Outcomes Measurement Information System (PROMIS). Medical Care.

[9] Cella D, Gershon R, Lai JS, Choi S (2007). The future of outcomes measurement: item banking, tailored short-forms, and computerized adaptive assessment. Quality Life Research.

[10] Varni JW, Stucky BD, Thissen D, DeWitt EM, Irwin DE, Lai JS (2010). PROMIS pediatric pain interference scale: An item response theory analysis of the pediatric pain item bank. The Journal of Pain.

[11] Yeatts KB, Stucky B, Thissen D, Irwin D, Varni JW, DeWitt EM (2010). Construction of the pediatric asthma impact scale (PAIS) for the Patient-Reported Outcomes Measurement Information System (PROMIS). Journal of Asthma.

[12] Irwin DE, Gross HE, Stucky BD, Thissen D, DeWitt EM, Lai JS (2012). Development of six PROMIS pediatrics proxy-report item banks. Health and Quality of Life Outcomes.

[13] Irwin DE, Stucky BD, Langer MM, Thissen D, DeWitt EM, Lai JS (2012). PROMIS pediatric anger scale: An item response theory analysis. Quality of Life Research.

[14] Walsh TR, Irwin DE, Meier A, Varni JW, DeWalt DA (2008). The use of focus groups in the development of the PROMIS pediatrics item bank. Quality of Life Research.

[15] Irwin D, Stucky B, Langer M, Thissen D, DeWitt E, Lai JS (2010). An item response analysis of the pediatric PROMIS anxiety and depressive symptoms scales. Quality of Life Research.

[16] Khanna D, Krishnan E, Dewitt EM, Khanna PP, Spiegel B, Hays RD (2011). The future of measuring patient-reported outcomes in rheumatology: Patient-Reported Outcomes Measurement Information System (PROMIS). Arthritis Care Research (Hoboken).

[17] Riley WT, Pilkonis P, Cella D (2011). Application of the National Institutes of Health Patient-Reported Outcome Measurement Information System (PROMIS) to mental health research. Journal of Mental Health Policy and Economics.

[18] Eisenstein EL, Diener LW, Nahm M, Weinfurt KP (2011). Impact of the Patient-Reported Outcomes Management Information System (PROMIS) upon the design and operation of multi-center clinical trials: a qualitative research study. Journal of Medical Systems.

[19] Raad voor de Nederlandse Taal en Letteren. (2003). *Variatie in het Nederlands: eenheid in verscheidenheid*.

[20] Cella, D. Center on Outcomes, R. a. E. (Ed.). (1997). *Manual of the Functional Assessment Of Chronic Illness Therapy (FACIT) measurement system (Version 4)*. Evanston. 10.1186/1477-7525-1-79 PMC317391 14678568

[21] Eremenco SL, Cella D, Arnold BJ (2005). A comprehensive method for the translation and cross-cultural validation of health status questionnaires. Evaluation Health Professions.

[22] Flaherty JA, Gaviria FM, Pathak D, Mitchell T, Wintrob R, Richman JA (1988). Developing instruments for cross-cultural psychiatric research. Journal of Nervous and Mental Disease.

[23] Forrest CB, Bevans KB, Tucker C, Riley AW, Ravens-Sieberer U, Gardner W (2012). Commentary: The Patient-Reported Outcome Measurement Information System (PROMIS) for children and youth: application to pediatric psychology. Journal of Pediatric Psychology.

[24] Varni JW, Thissen D, Stucky BD, Liu Y, Gorder H, Irwin DE (2012). PROMIS(R) parent proxy report scales: An item response theory analysis of the parent proxy report item banks. Quality of Life Research.

[25] Varni JW, Magnus B, Stucky BD, Liu Y, Quinn H, Thissen D (2014). Psychometric properties of the PROMIS (R) pediatric scales: Precision, stability, and comparison of different scoring and administration options. Quality of Life Research.

